# A case-control study on risk factors for early-onset respiratory tract infection in patients admitted in ICU

**DOI:** 10.1186/1471-2466-7-12

**Published:** 2007-09-21

**Authors:** Teresa C Cardoso, Luís M Lopes, António H Carneiro

**Affiliations:** 1 Intensive Care Unit, Hospital Geral de Santo António, Porto, Portugal, Unidade de Cuidados Intensivos Polivalente, Hospital Geral de Santo António, Largo Prof. Abel Salazar, 4099-001 Porto, Portugal; 2 Intensive Care Unit, Hospital Geral de Santo António, Porto, Portugal, Emergency Department, Hospital de São João, Alameda Prof. Hernâni Monteiro, 4200-319 Porto, Portugal

## Abstract

**Background:**

Respiratory tract infections are common in intensive care units (ICU), with incidences reported from 10 to 65%, and case fatality rates over 20% in pneumonia. This study was designed to identify risk factors for the development of an early onset respiratory tract infection (ERI) and to review the microbiological profile and the effectiveness of first intention antibiotic therapy.

**Methods:**

Case-control, retrospective clinical study of the patients admitted to the Intensive Care Unit (ICU) of our hospital, a teaching and tertiary care facility, from January to September 2000 who had a respiratory tract infection diagnosed in the first 5 days of hospital stay.

**Results:**

Of the 385 patients admitted to our unit: 129 (33,5%) had a diagnosis of ERI and 86 patients were admitted to the control group. Documented aspiration (adjusted OR = 5,265; 95% CI = 1,155 – 24,007) and fractured ribs (adjusted OR = 12,150; 95% CI = 1,571 – 93,941) were found to be independent risk factors for the development of ERI (multiple logistic regression model performed with the diagnostic group as dependent variable and adjusted for age, sex, SAPS II, documented aspiration, non-elective oro-tracheal intubation (OTI), fractured ribs, pneumothorax and pleural effusion).

A total of 78 organisms were isolated in 61 patients (47%). The normal flora of the upper airway (*Streptococcus pneumoniae, Staphylococcus aureus, Haemophilus influenza *and *Moraxella catharralis*) accounted for 72% of all isolations achieved, polimicrobian infections were responsible for 25% of all microbiological documented infections. First intention treatment was, in 62% of the patients, the association amoxacillin+clavulanate, being effective in 75% of the patients to whom it was administered.

The patients with ERI needed more days of OTI (6 vs 2, p < 0,001) and mechanical ventilation (6 vs 2, p < 0,001) and had a longer ICU (7 vs 2, p < 0,001) and hospital length of stay (17 vs 11, p = 0,018), when compared with controls.

**Conclusion:**

In this study documented tracheobronchial aspiration and fractured ribs were identified as independent risk factors for ERI. Microbiological profile was dominated by sensitive micro-organisms. The choice amoxacilin+clavulanate revealed to be a good option with an effectiveness rate of 77% in the patients in whom it was used.

## Background

Respiratory tract infections are common in intensive care units (ICU), with incidences reported from 10 to 65%, and case fatality rates over 20% in pneumonia [1].

Respiratory tract infections can be divided in early and late onset, according to the time of diagnosis after ICU or hospital admission [1,2]. This differentiation is very important since that it has important implications: early onset respiratory tract infections are considered to be mainly caused by antibiotic-sensitive pathogens and late onset respiratory tract infections by antibiotic-resistant micro-organisms select by previous antibiotic therapy or hospital acquired flora, thus requiring different therapeutic approaches.

In the last decade several studies have been developed in order to identify risk factors for the development of respiratory tract infections in the intensive care environment [3], but despite all the advances in the diagnostic, treatment and prevention, respiratory tract infections are still a major cause of death due to hospital-acquired infection [1,2].

The purpose of this study is to identify risk factors for the development of early respiratory tract infection in an intensive care environment and to review the microbiological profile and the effectiveness of first intention antibiotic therapy in early respiratory tract infections (ERI), in an attempt to obtain useful data that would allow identification of patients at risk (and thus implement preventive measures) and to initiate early and adequate antibiotic therapy (to improve the prognosis).

## Methods

This was a case-control, retrospective clinical study, done at the Intensive Care Unit of Hospital Geral de Santo António (600 beds), a twelve bed mixed ICU at an university-affiliated urban teaching hospital, located in Porto, Portugal, during a 9 months period (between January and September 2000).

At the time the hospital didn't require any Ethical Committee approval for purely observational studies. Informed consent was waived since this was an observational study without any deviation from the current medical practice.

During that period all patients admitted to the ICU, with a diagnosis of a respiratory tract infection made over the first five days of hospital stay were defined as having an early onset respiratory tract infection (ERI) and thus as the study group.

Tracheobronchitis was defined by purulent tracheo-bronchial secretions, with fever (> 38,5°C, axillar) and leukocytosis (> 10.000), and pneumonia by the 3 mentioned criteria and "new" and persistent pulmonary infiltrate.

The control group were all patients admitted to ICU over the same period but without a diagnosis of respiratory tract infection throughout their ICU stay.

Patients with an ICU stay of less than 24 hours, admitted with community-acquired respiratory tract infection or who developed a respiratory tract infection after 5 days of hospital stay, were excluded.

For each patient the following information was recorded: demographic characteristics (age, sex), admission category (medical, trauma, elective surgery, non-elective surgery), data of hospital and ICU admission, date of ICU and hospital discharge, the SAPS II for the first 24 hours of ICU admission and the ICU and hospital outcomes. For the calculation of SAPS II laboratory and clinical data not measured were assigned the value score of zero. To determine neurological status patients receiving sedative drugs were assigned the Glasgow Coma Score (GCS) measured or estimated before sedation.

It was also registered length of oro-tracheal intubation, mechanical ventilation and ICU stay, comorbidities, risk factors for the development of respiratory tract infections, mechanical alterations of ventilation, microbiological profile obtained and first intention antibiotic therapy.

The presence of underlying disease (metastatic cancer, haematological malignancy, acquired immunodeficiency syndrome – AIDS) were recorded using SAPS II definitions [4]. Cirrhosis, chronic heart failure, chronic pulmonary failure was defined using Acute Physiology and Chronic Health Evaluation II [5]; chronic renal failure as the need of chronic renal support or history of chronic renal insufficiency with a serum creatinine level over 2 mg/dl; human immunodeficiency virus (HIV) status – HIV infection without complications defining AIDS; immuno-compromised state by either administration in the 12 months prior to ICU admission of chemotherapy, radiation therapy or the equivalent to 0,2 mg/Kg/day prednisolone for at least 3 months or 1 mg/Kg/day for a week in the previous 3 months to ICU admission.

The following were considered as possible risk factors for tracheobronchial aspiration: altered conscious (8<GCS<15), coma (9<GCS), intoxication, seizures, vomiting and respiratory or cardiopulmonary arrest, and thus for the development of ERI.

The presence of mechanical alterations of ventilation (spinal cord injury, recent abdominal injury or surgery, fractured ribs, pulmonary contusion, pneumothorax, pleural effusion and atelectasy) that would contribute to a deficient clearance of bronchial secretions might also play a role in the development of ERI.

The microbiological profile of the products collected in the first 72 hours and the antimicrobial therapy (its efficacy, failure or modification according to the microorganism sensibility) were also reviewed.

We considered antibiotic failure when there was poor clinical response defined as the persistence and/or worsening of the clinical signs or symptoms after 72 h of treatment.

Descriptive analyses is made of background variables (gender, age), ICU variables (SAPS II, length of OTI, mechanical ventilation and ICU stay) and hospital length of stay. Categorical variables were described as absolute frequencies (n) and relative frequencies (%); median and percentiles were used for continuous variables. Pearson Qui Square and Mann-Whitney test were used for comparisons.

Multiple logistic regression was performed with the diagnostic group as dependent variable and age, sex, SAPS II, documented aspiration, non-elective OTI, fractured ribs, pneumothorax and pleural effusion as independent variables. Stepwise Forward method was used with an entry criteria of p < 0,05 and a removal criteria of p < 0,1.

Statistical significance was considered at p < 0,05. SPSS^® ^15.0 was used for statistical analysis.

## Results

During the study period 385 patients were admitted to our unit, of those 215 were admitted in the study (Table 1),170 were excluded (49, due to an ICU stay of less than 24 hours and 121 patients because they were admitted with the diagnosis of community-acquired respiratory tract infection or developed a respiratory tract infection after 5 days of hospital stay). The median age of the patients was 48 years, and the median SAPS II score was 41. Sixty-four (30%) were women and 151 (70%) were men.

**Table 1:** Baseline characteristics of the study population

	**Total n = 215**	**Control group n = 86**	**Study group n = 129**	**p**
**Sex **n (%)				
Female	64 (30)	32 (37)	32 (25)	0,051
Male	151 (70)	54 (64)	97 (75)	
**Age **median (IQR)	48 (27–65)	43 (26–59)	51 (29–67)	0,092
**SAPS II **median (IQR)	41 (33–53)	42 (27–55)	40 (33–51)	0,901
**Diagnosis on admission **n (%)				
Trauma	93 (43)	35 (41)	58 (45)	
Medical	93 (43)	33 (38)	60 (46)	
Urgent surgery	28 (13)	17 (20)	11 (9)	
Elective surgery	1 (0)	1 (1)	0 (0)	

IQR – Inter-quartil range

A hundred and twenty-nine patients (33,5%) had a diagnostic of ERI (of those 57 had pneumonia – 14,8%) and 86 were admitted to the control group. No significant differences were seen between the two groups regarding sex, age and SAPS II (Table 1).

A univariate logistic forward stepwise regression analysis with ERI as the dependent factor and each of the comorbidities as independent variables didn't show any significant association between theme.

The same analysis for the previous mentioned risk factors showed an association of ERI with documented tracheal aspiration, non-elective OTI, fractured ribs, pneumothorax and pleural effusion (Table 2).

**Table 2:** Possible risk factors for the development of early-respiratory tract infection

	**Total **n = 215	**Control group **n = 86	**Study group **n = 129	**OR**	**95% CI**	**p**
**Documented aspiration, n (%)**						
No	200 (93)	84 (98)	116 (90)	1		
Yes	15 (7)	2 (2)	13 (10)	4.707	[1.035–21.413]	0,029
**Non-elective OTI, n (%)**						
No	64 (30)	34 (40)	30 (23)	1		
Yes	151 (70)	52 (60)	99 (77)	2.158	[1.190–3.911]	0,048
**Fractured ribs, n (%)**						
No	199	85 (99)	114 (88)	1		
Yes	16	1 (1)	15 (12)	11.184	[1.449–86.327]	0,004
**Pneumothorax, n (%)**						
No	201	84 (98)	117 (91)	1		
Yes	14	2 (2)	12 (9)	4.308	[0.939–19.754]	0,042
**Pleural effusion, n (%)**						
No	185	80 (93)	105 (81)	1		
Yes	30	6 (7)	24 (19)	3.048	[1.190–7.807]	0,016

OTI – oro-tracheal intubation. OR – odds ratio. CI – Confidence interval

In the multiple logistic regression model performed with the diagnostic group as dependent variable and adjusted for age, sex, SAPS II, documented aspiration, non-elective OTI, fractured ribs, pneumothorax and pleural effusion as independent variables, documented aspiration (adjusted OR = 5,265; 95% CI = 1,155 – 24,007) and fractured ribs (adjusted OR = 12,150; 95% CI = 1,571 – 93,941) were found to be independent risk factors for the development of ERI.

A total of 78 organisms were isolated in 61 patients (isolation rate 47%) (Table 3).

**Table 3:** Microbiological profile found according to the day of specimen collection

**MICRORGANISM (n = 78))**	**Day 1**	**Day 2**	**Day 3**	**Day 4**	**Day 5**
***Haemophilus influenza *(26)**	**7**	**6**	**6**	**4**	**3**
***MSSA *(15)**	**4**	**4**	**2**	**2**	**3**
***Streptococcus pneumoniae *(14)**	**4**	**6**	**1**	**1**	**2**
***Moraxella catarrhalis *(1)**		**1**			
*MRSA *(2)				1	1
*Pseudomonas aeruginosa *(6)		1	3		2
*E. coli *(3)	2	1			
*Acinectobacter *(1)		1			
*Serratia marcenses *(2)		1		1	
*Proteus mirabilis *(1)			1		
*Klebsiella pneumoniae *(2)				1	1
*Enterobacter cloacae *(2)		1		1	
*Candida albicans *(2)			1		1
*Candida Kruzi *(1)				1	
Polymicrobial (15)	2	6	2	3	2

In early-onset pneumonia the microbiological profile was dominated by MSSA (37%), followed by Haemophilus influenza (28%) and Streptococcus pneumoniae (22%); 6 of these patients (10%) had a polimicrobian infection.

In early-onset tracheobronchitis: Haemophilus influenza represented 37% of all isolations, followed by Streptococcus pneumoniae (15%) and MSSA (7%); polimicrobian infections were present in 9 patients (10%).

In Table 4 it is shown the more frequently used antibiotic treatments. First intention antibiotic therapy was considered effective in 74% of the patients and modified according to the microbiological profile in 7% and due to failure in 19% of the cases.

**Table 4:** First intention antibiotic therapy

**ANTIBIOTHERAPY (%)**	**Total n(%) **129 (100)	**Failure n(%) **24 (19)	**Directed n(%) **9 (7)
Amoxacillin+calvulanate (62)	80 (100)	18 (23)	2 (3)
Amoxacillin+calvulanate+aminoglycoside (7)	9 (100)	1 (11)	3 (33)
3^rd ^generation cephalosporin (8)	10 (100)	3 (30)	1 (10)
Carbapenem (8)	10 (100)	0 (0)	2 (20)
Other (15)	20 (100)	2 (10)	1 (5)

(Total) Number of patients in whom it was used. (Failure) Number of patients in whom it was modified due to failure. (Directed) Number of patients in whom it was modified according to the microbiological profile.

The study group needed more days of OTI and mechanical ventilation and had a longer ICU and hospital length of stay (Table 5) when compared to the control group.

**Table 5:** Days of oro-tracheal intubation (OTI), mechanical ventilation ICU and hospital length of stay (LOS) in the study and control groups

Days	Total	Control group	Study group	P
OTI (median, IQR)	4 (2–8)	2 (1–3)	6 (4–11)	<0,001
Mechanical ventilation (median, IQR)	4 (2–7)	2 (1–3)	6 (3–10)	<0,001
ICU LOS (median, IQR)	4 (2–8)	2 (1–3)	7 (4–10)	<0,001
Hospital LOS (median, IQR)	15 (7–31)	11 (3–36)	17 (9–31)	0,018

IQR – Inter-quartil range.

## Discussion

In the study period the incidence of early respiratory tract infection among patients admitted to our ICU was 34%.

A clinical diagnosis of respiratory infection was used and the incidence of pneumonia found (15%) was similar [1,6,9] or even lower [10-14] to what has been described by others using also clinical criteria and similar to the ones who based the diagnosis on bronchoscopic techniques [12,13].

The definition of early respiratory tract infection itself is controversial, with time definitions in literature that range from 48 hours to 7 days [1,2,10-13]. And the starting point is also controversial with studies considering OTI [10,12], ICU admission [2] or hospital admission time [1,11]. For the purpose of this study the first five days of hospital stay were considered in an attempt to by-pass the role of colonization with the usual hospital flora.

Fractured ribs and documented tracheo-bronchial aspiration were found to be independent risk factors for the development of ERI. This should lead us to the prompt recognition and resolution of these problems with analgesia and physiotherapy in the first case and prophylactic antibiotic therapy in the second, as already suggested by previous studies [10], attempting to prevent the development of a respiratory tract infection.

We achieved an isolation rate of 47%. Among these patients the usual oral flora represented 72% of the total, polimicrobian infections were present in 23% also suggesting that aspiration might play a major role in the pathogenesis of ERI.

None of our patients was under antibiotic therapy previously, which can explain the high rate of *Haemophilus influenza*; 34% were trauma patients probably justifying *Staphylococcus aureus MS *has the third agent.

When looking at the microbiological profile in Table 3 it is not possible to find a clear cut at a specific time point where it is possible to say it changes from antibiotic sensitive microrganisms to antibiotic resistant. The overall microbiological profile is dominated by sensitive micro-organisms suggesting that in this ICU respiratory tract infections diagnosed in the first five days of hospital admission should be treated as early-onset.

Antibiotic resistant organisms were identified in patients with multiple comorbidities (particularly structural lung disease, alcohol abuse and smoking) and patients transferred from other hospitals which usual flora wasn't known.

In the literature, among similar studies that also used the clinical definition of early onset pneumonia, the majority [6,9-13] also showed a microbiological profile dominated by antibiotic sensitive organisms but there are similar studies [1,15] where it is dominated by antibiotic resistant organisms, suggesting that the hospital environment might play an important role in the aetiology.

First intention antibiotic therapy was empirical in every patient and initiated as soon as the diagnose was made. This option is fully justified by the severity of the pathology, the low sensitivity and specificity of tracheo-bronchial aspirate for microbiological assessment (the usual procedure in the ICU of this study) and the delay in obtaining the results, along with the knowledge that any delay in initiating appropriate therapy determines a worse prognosis [2,14].

Overall the initial antibiotic choice had to be changed in 33 patients (26%), a lower rate when compared to a multicenter Spanish study published by Alvarez [16], that accessed the frequency of and the reasons for changing empiric antibiotics used in the treatment of pneumonia acquired in the ICU.

Antibiotic therapy was changed according to the microbiological profile in 9 patients (39%), either to broaden or narrow the initial antibiotic choice according to the antibiogram and due to failure in 24 patients (61%); when comparing these results to the one's from the Spanish study [16] we see an inversion in the proportion of patients that had their therapy changed according to antibiogram and those due to therapy failure, this can be justified by the different isolation rate in both studies (much higher in the Spanish case).

According to the recommendations of ATS [2], first intention antibiotic therapy was, in 70% of the patients, amoxacilin+clavulanate or a third generation cephalosporin, which revealed to be a good option, with a failure rate of 23% in the patients to whom it was administered.

## Conclusion

In this study documented tracheobronchial aspiration and fractured ribs were identified as independent risk factors for ERI.

Knowing the expected microbiological profile is necessary to initiate prompt and effective antibiotic therapy as soon as the clinical diagnosis of ERI is made. In this study amoxacilin+clavulanate revealed to be a good option with an effectiveness rate of 77% in the patients in whom it was used.

Collection of clinical specimens for microbiological study is important to identifying resistant strains and guide alternative antibiotherapy.

## Key messages

• We identify as risk factors for the development of early-onset respiratory tract infections fractured ribs and documented tracheal aspiration.

• Early-onset respiratory tract infection was defined as developing in the first five days of hospital admission and the microbiological profile was dominated by sensitive microrganisms.

## Competing interests

The author(s) declare that they have no competing interests.

## Authors' contributions

All authors created and designed the study and collected the data. TC and AC wrote the final manuscript.

## Pre-publication history

The pre-publication history for this paper can be accessed here:


